# Supplementary material for: Unexpected Genetic Diversity of Two Novel Swine MRVs in Italy
Source: Viruses. 2020 May 22;12(5):574. doi: 10.3390/v12050574 (PMC7290992; doi:10.3390/v12050574)
Supplement: Supplementary file 1 [file viruses-12-00574-s001.pdf]

Unexpected genetic diversity of two novel swine MRVs in Italy

Lara Cavicchio[1§], Luca Tassoni[1§], Gianpiero Zamperin[2], Mery Campalto[1], Marilena Carrino[1], Stefania Leopardi[2], Paola De Benedictis[2] and Maria Serena Beato[1*]

[1] Diagnostic Virology Laboratory, Department of Animal Health, Istituto Zooprofilattico Sperimentale delle Venezie (IZSVe), Viale dell'Università 10, 35020, Legnaro, Padua, Italy.
[2] OIE Collaborating Centre for diseases at the animal/human interface, Istituto Zooprofilattico Sperimentale delle Venezie (IZSVe), Viale dell'Università 10, 35020, Legnaro, Padua, Italy.
§ These authors have equally contributed
* Correspondence: msbeato@izsvenezie.it

Supplementary Table S1. Primers used to amplify each terminal gene segment.

Primer ID	Primer sequence (5′- 3′)	Target gene	Reference
**S1-F1**	5′-GCTATTCGTACTGATGTCTGAGCTT-3′	S1	Wang et al. 2015 [34]
**S1 5′ UTR Rev**	5′-CCCTTTCATTTGTKCCGACA-3′		Primer designed for the present study
**S1 3′ UTR For**	5′-GCGGTATTGACAGTTGGACAAGG-3′		Primer designed for the present study
**S1-R1**	5′-GATGAATCGCCGTCGTGCCGG-3′		Wang et al. 2015 [34]
**S2-F1**	5′-GCTATTCGCTGGTCAGTTATGG-3′	S2	Wang et al.2015 [34]
**S2 5′ UTR Rev**	5′-CCACTCCACATATCYTCRTCC-3′		Primer designed for the present study
**S2 3′ UTR For**	5′-CGCAGYTTATTCGTYATGCC-3′		Primer designed for the present study
**S2-R1**	5′-GATGAATGTGTGGTCAGTCGTGAA-3′		Wang et al. 2015 [34]
**S3-F1**	5′-GCTAAAGTCACGCCTGTTGTCGT-3′	S3	Wang et al. 2015 [34]
**S3 5′ UTR Rev**	5′-TGTAGCCCAGCCATACCCAT-3′		Primer designed for the present study

**S3 3′ UTR For**	5′-GATGATTAAGCGCCACCCACCACC-3′		Primer designed for the present study
**S3-R1**	5′-GCGAGGAAACGGCAGAAGC-3′		Wang et al. 2015 [34]
**S4-F1**	5′-GCTATTTTTGCCTCTTCCCAAACGT-3′	S4	Wang et al. 2015 [34]
**S4 5′ UTR Rev**	5′-CACCCTCCAGCTTCACTCCA-3′		Primer designed for the present study
**S4 3′ UTR For**	5′-GGAGTGAAGCTGGAGGGTGA-3′		Primer designed for the present study
**S4-R1**	5′-GATGAATGAAGCCTGTCCCACGTC-3′		Wang et al.2015 [34]
**M1_F1**	5′-GCTATTCGCGGTCATGGCTTACATC-3′	M1	Wang et al.2015 [34]
**M1 5′ UTR Rev**	5′-ACCCGACCCGCCTCTTTGCC-3′		Primer designed for the present study
**M1 3′ UTR For**	5′-CGGTTGTGAGGATGGGTTCG-3′		Primer designed for the present study
**M1_R2**	5′-GATGAAGCGCGTACGTAGTCTTAGC-3′		Wang et al.2015 [34]
**M2_F1**	5′-GCTAATCTGCTGACCGTTACTCTGC-3′	M2	Wang et al. 2015 [34]
**M2 5′ UTR Rev**	5′-GCCACTCTCAGGTCAATCTCCCAC-3′		Primer designed for the present study
**M2 3′ UTR For**	5′-CCAATTCAAGGTCAGCTCGC-3′		Primer designed for the present study
**M2_R2**	5′-GATGATTTGCCTGCGTCCCTTAACC-3′		Wang et al. 2015 [34]
**M3_F1**	5′-GCTAAAGTGACCGTGGTCATGGCTT-3′	M3	Wang et al. 2015 [34]
**M3 5′ UTR Rev**	5′-CCCAAATAGCATCAGCTCCTCA-3′		Primer designed for the present study
**M3 3′ UTR For**	5′-GCTCAATCATGCAGTCTGGACA-3′		Primer designed for the present study

**M3_R2**	5′-TGAATGGGGGTCGGGAAGGCTTAAG-3′		Wang et al. 2015 [34]
**L1_F1**	5′-GCTACACGTTCCACGACAATGTCATC-3′	L1	Wang et al. 2015 [34]
**L1 5′ UTR Rev**	5′-GACCAGATATGTGACGGCAGA-3′		Primer designed for the present study
**L1 3′ UTR For**	5′-GCTCAGACACTATTCATGGCAAAGA-3′		Primer designed for the present study
**L1_R5**	5′-GATGAGTTGACGCACCACGG-3′		Wang et al. 2015 [34] – modified
**L2 3′ UTR For**	5′-GCGTAGACATCAGACCAACGGC-3′	L2	Primer designed for the present study
**L2_R4**	5′-GATGAATTAGGCGCGCTCACGAGGG-3′		Wang et al. 2015 [34] – modified
**L3_F1**	5-′GCTAATCGTCAGGATGTGTATAAGA-3′	L3	Wang et al. 2015 [34] – modified
**L3 5′ UTR Rev**	5′-GCATTATTGATACCAGTGTCT-3′		Primer designed for the present study
**L3 3′ UTR For**	5′-CACCAACGAGCATCCCATCCG-3′		Primer designed for the present study
**L3_R4**	5′-TGAATTGGCCCAACTAGCATCGAG-3′		Wang et al. 2015 [34]

Material and Methods

1.2 Virus isolation in cell cultures

Cell monolayers were washed three times with PBS and inoculated with faeces homogenates previously filtered with a 0.45 μm filter (Merck Millipore, Burlington, Massachusetts, USA) and post-inoculation medium: MEM with phosphor tryptose broth (0.3% v/v), yeast extract (0.02% v/v) and trypsin 5 μg/ml (Sigma Aldrich, St. Louis Missouri, USA). Adsorption was carried out at +37 °C and 5% CO_2 for two hours. Subsequently, cell monolayers were observed daily for six days to detect any change in cell morphology and any cytopathic effect (CPE). If CPE was not observed during the first cell passage, other two blind passages were carried out before ruling out the absence of virus growth. Briefly, EM was conducted on repeatedly frozen and thawed cell cultures and clarified by a two-step centrifugation (2,500 x *g*) at 4 °C for 30 min and 7,000 x *g* at 4 °C for 30 min. An aliquot of 85 μL of the supernatant was ultracentrifuged for 15 min in a Beckman Airfuge, using an A-100 rotor, at 20 psi (125,000 x *g*). The grids were stained using a 2% sodium phosphotungstate solution in distilled water (pH 6.8) for at least 3 min. The dried grids were observed using a TEM Philips operating at 80 kV, at a

magnification of 19,000-45,000. A minimum of 20 viral particles were measured at a magnification of 36,000 and statistically analysed with Soft-imaging software analySIS 2.1 (GmbH_ 1996).

Results

1.2 Identification of two novel MRVs in one single swine farm in North Eastern Italy

Two MRV positive faeces samples of case 16DIAPD52154/2016 (n. 2 and 4) presented CPE on day six p.i. during the second passage (Figure 1 A), two samples (n. 3 and 6) on day three p.i. during the third passage, one sample (n.1) on day two p.i. during the fourth passage.

The beginning of the CPE was observed on day three p.i. during the first, third and fourth passages for faeces samples n. 1, 5 and 3 (Figure 1 B) of case 18DIAPD90178/2018, respectively and on day six p.i. during the second passage for sample n. 6. Interestingly, 18DIAPD90178/2018 sample n. 8, although positive for virus isolation, never presented an evident CPE.

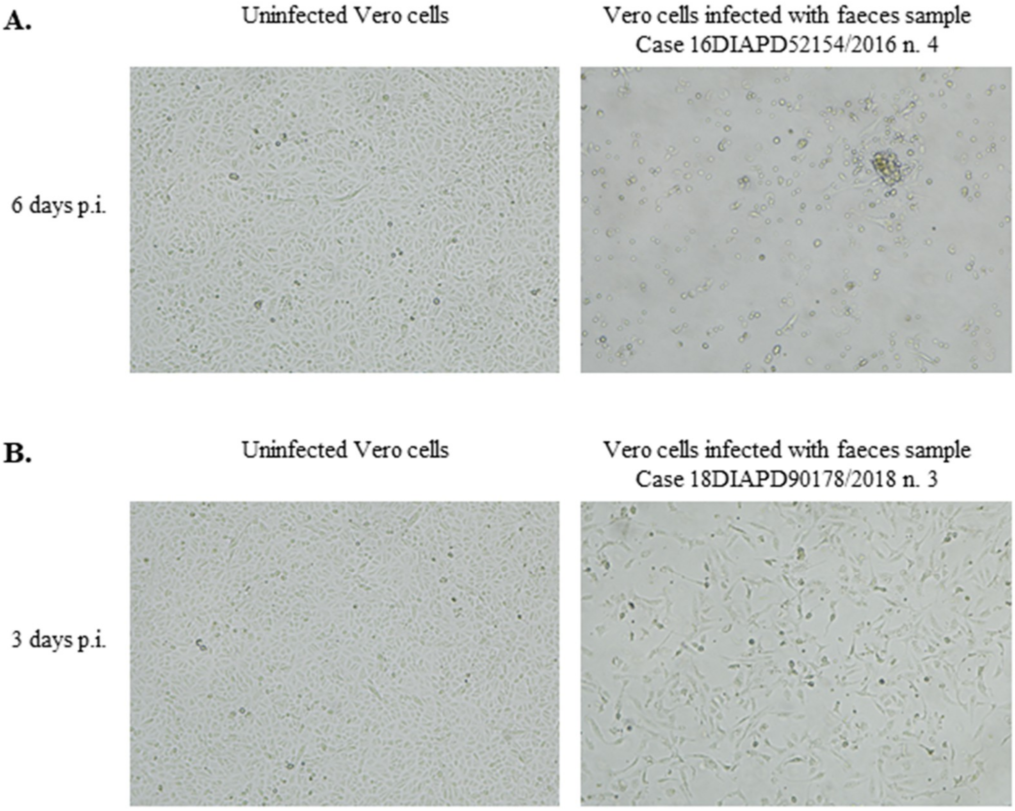

Supplementary Figure S1. Uninfected Vero cells and cytopathic effects observed in Vero cells infected with faeces samples and virus strains. A: case 16DIAPD52154/2016 infected with faeces sample n.4 at six days p.i.; B: Vero cells infected with faeces samples of case 18DIAPD90178/2018 n 3 at 3 days p.i.

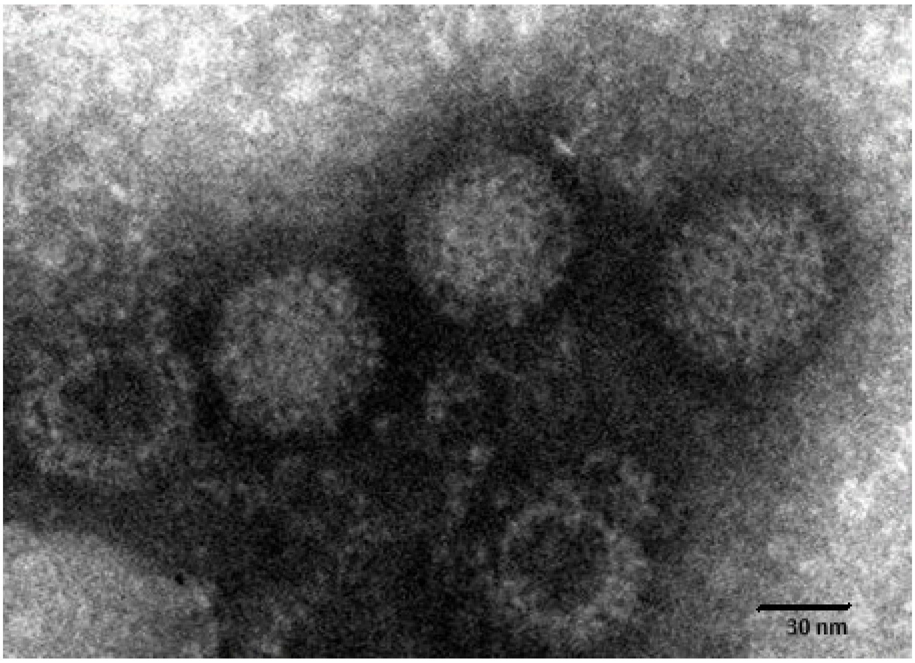

Supplementary Figure S2. Electron microscopy picture of *Reoviridae* particles isolated from MRV3/swine/Ita/2016 infected Vero cell cultures.

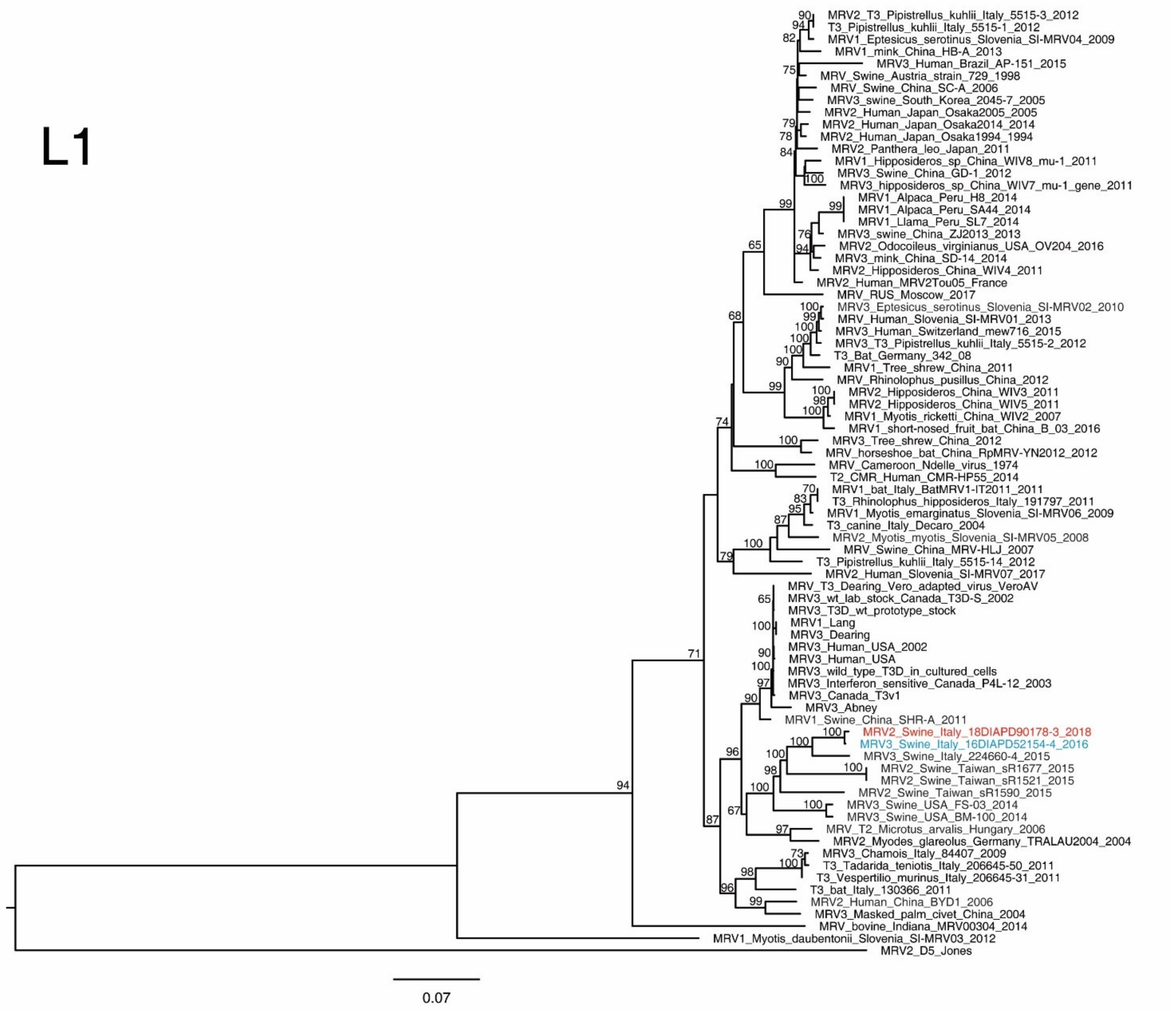

Supplementary Figure S3. Phylogenetic analysis based on the L1 nucleotide sequence of the MRV2/swine/Italy/90178-3/2018 (red) and MRV3/swine/Italy/52154-4/2016 (light blue) strains.

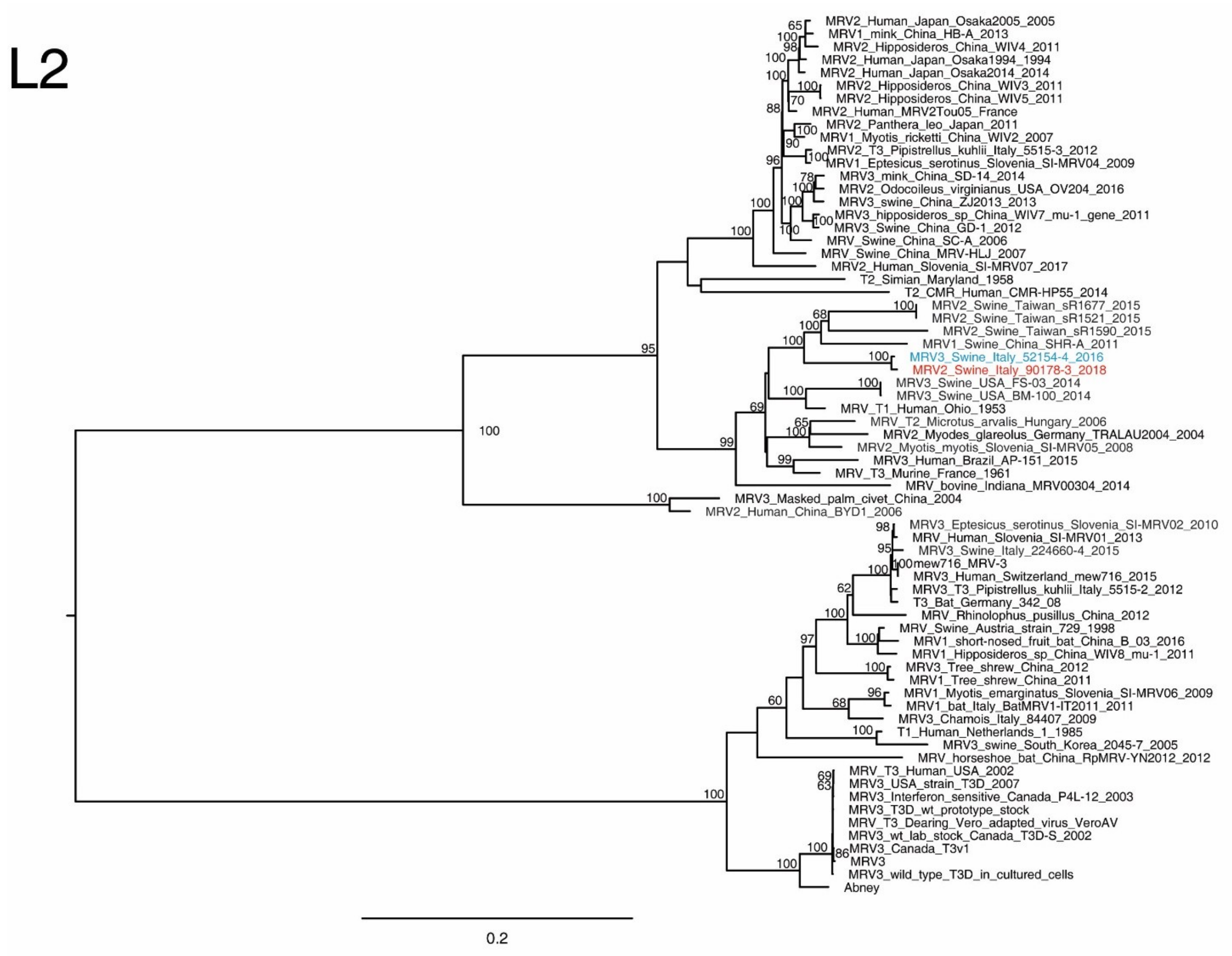

Supplementary Figure S4. Phylogenetic analysis based on the L2 nucleotide sequence of the MRV2/swine/Italy/90178-3/2018 (red) and MRV3/swine/Italy/52154-4/2016 (light blue) strains.

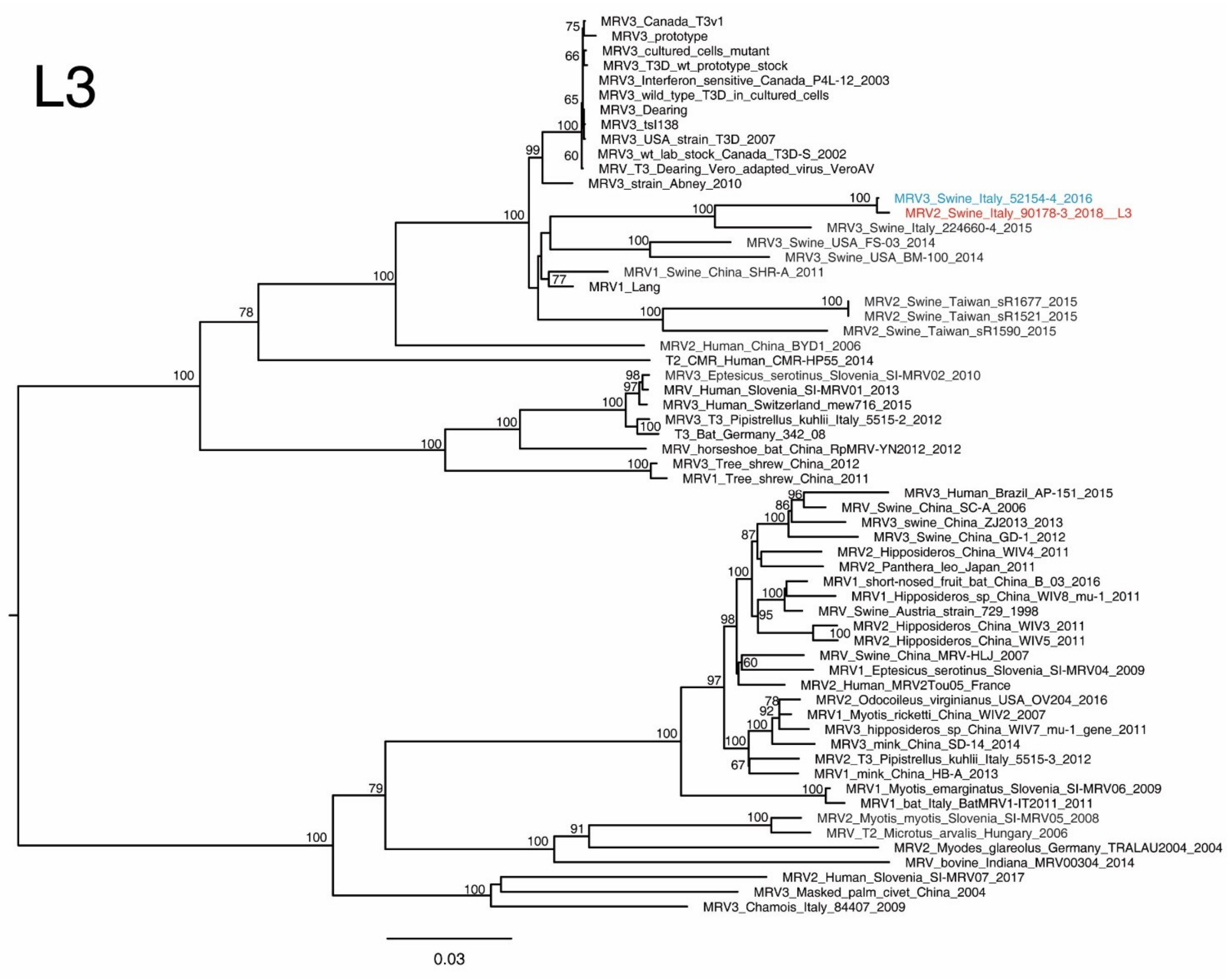

Supplementary Figure S5. Phylogenetic analysis based on the L3 nucleotide sequence of the MRV2/swine/Italy/90178-3/2018 (red) and MRV3/swine/Italy/52154-4/2016 (light blue) strains.

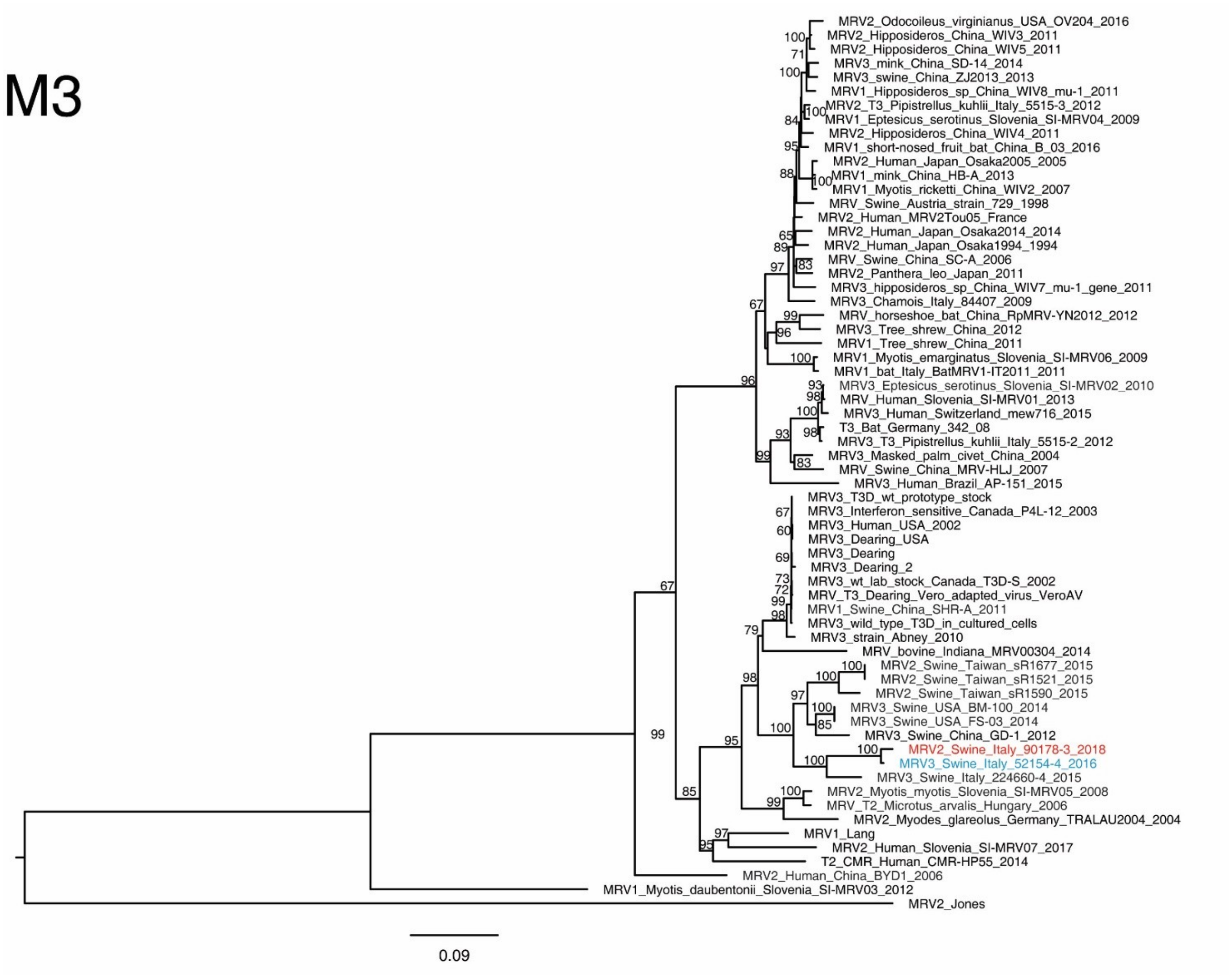

Supplementary Figure S6. Phylogenetic analysis based on the M3 nucleotide sequence of the MRV2/swine/Italy/90178-3/2018 (red) and MRV3/swine/Italy/52154-4/2016 (light blue) strains.

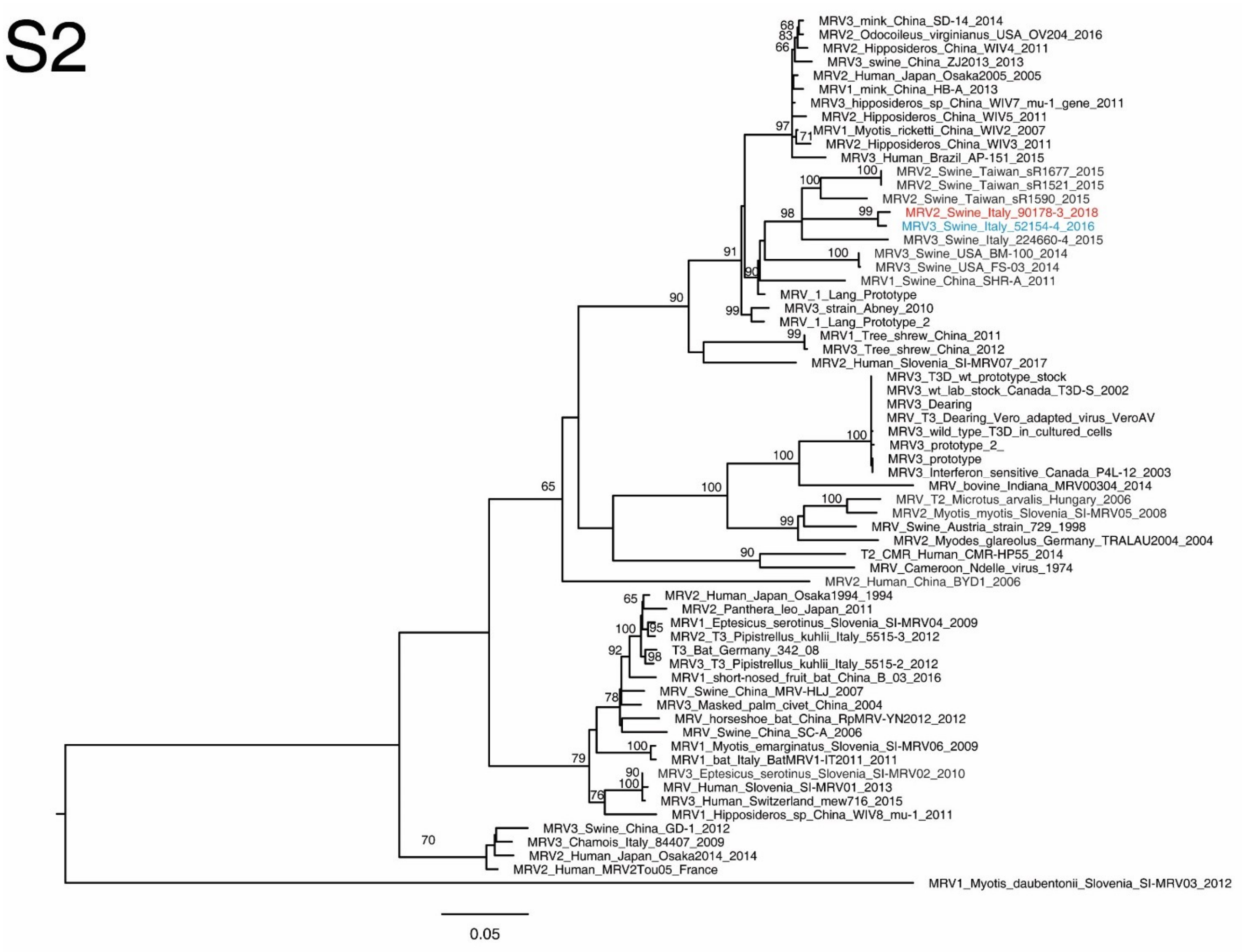

Supplementary Figure S7. Phylogenetic analysis based on the S2 nucleotide sequence of the MRV2/swine/Italy/90178-3/2018 (red) and MRV3/swine/Italy/52154-4/2016 (light blue) strains.

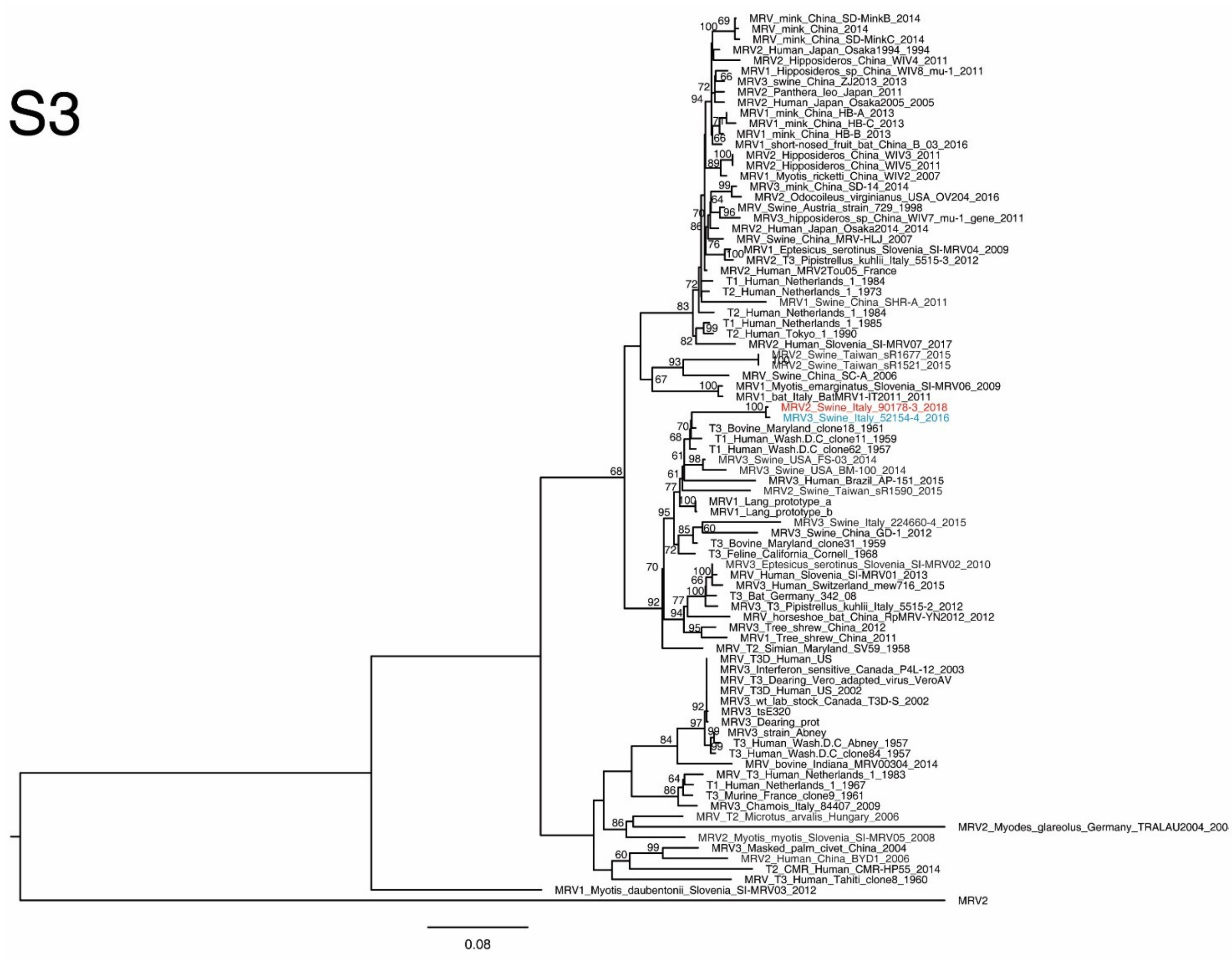

Supplementary Figure S8. Phylogenetic analysis based on the S3 nucleotide sequence of the MRV2/swine/Italy/90178-3/2018 (red) and MRV3/swine/Italy/52154-4/2016 (light blue) strains.

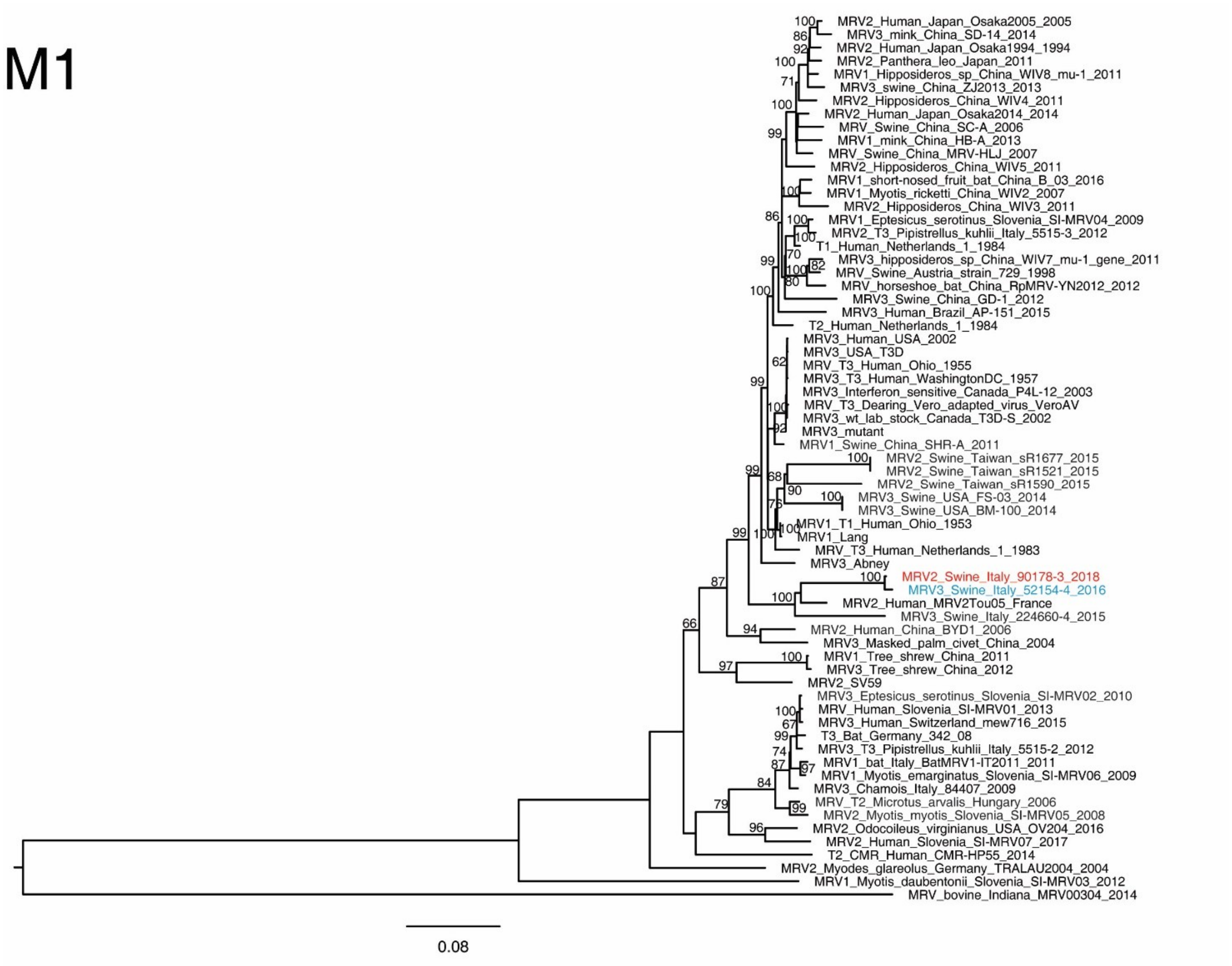

Supplementary Figure S9. Phylogenetic analysis based on the M1 nucleotide sequence of the MRV2/swine/Italy/90178-3/2018 (red) and MRV3/swine/Italy/52154-4/2016 (light blue) strains.
